# Preferences for Decision-Making Style and Knowledge of and Attitudes To Recovery in Mental Health Professionals Working in Inpatient and Outpatient Settings in Routine Mental Health Practice: An Exploratory Cross-Sectional Study in the Danish Mental Health Services

**DOI:** 10.1007/s10488-025-01472-9

**Published:** 2025-09-13

**Authors:** Lisa Korsbek, Stine Bjerrum Moeller, Marie Bonde, Rikke Amalie Agergaard Jensen

**Affiliations:** 1 Mental Healthcare Services, Region of Southern Denmark, Mental Health Unit Odense-Svendborg, Odense, Denmark; 2 Research unit of Psychiatry, Department of Clinical Research, University of Southern Denmark, Odense, Denmark; 3 Mental Healthcare Services, Department of Trauma and Torture Survivors, Region of Southern Denmark, Vejle, Denmark; 4 Department of Psychology, University of Southern Denmark, Danish Center of Psychotraumatology, Odense, Denmark; 5 Medical School, Faculty of Health Science, University of Southern Denmark, Odense, Denmark; 6 Department of Regional Health Research, Research Unit Mental Health South West, University of Southern Denmark, Odense, Denmark; 7 Mental Healthcare Services, Center for Involvement of Relatives, Region of Southern Denmark, Vejle, Denmark

**Keywords:** Mental health, Clinical decision-making, Shared decision-making, Decision-making preferences, Recovery, Recovery knowledge and attitudes

## Abstract

In mental health care, shared decision making (SDM) is a central part of the recovery paradigm. However, implementing SDM can be challenging, and professionals may prefer different decision-making styles. This study explored preferences for decision-making style and examined their association with knowledge of and attitudes to recovery among mental health professionals in routine hospital-based services. An exploratory cross-sectional survey was conducted among mental health professionals (*N* = 552) in hospital-based services in one of Denmark’s five regions. Preferences for decision-making style were measured using the Clinical Decision Making Style Scale – Staff Questionnaire, while professionals’ knowledge of and attitudes to recovery were assessed using the Recovery Knowledge Inventory. Although the majority of participants (72.4%) preferred a shared decision-making style, there were differences in preferences based on profession, work experience, and setting. One in five reported having received SDM training, and fewer reported having access to decision-support tools. Indications of differences in knowledge of and attitudes to recovery between professionals’ preferences for decision-making styles were found: those who preferred a shared or active style seemed to score higher on the RKI compared to those who preferred a passive, clinician-led style. While descriptive in nature, the findings suggest patterns in decision-making preferences that may help inform future implementation efforts. The results also suggest a potential alignment between endorsement of shared- or active decision-making styles and recovery-oriented values. Further research is needed to investigate how preferences translate into actual clinical practice and how knowledge about and attitudes to recovery may be operationalized as recovery-oriented care.

Clinical decision-making (CDM) has been defined as a “contextual, continuous, and evolving process, where data are gathered, interpreted, and evaluated in order to select an evidence-based choice of action” (Tiffen et al., 2014, p. 401). In the literature, three central models of CDM, characterized by varying levels of patient involvement, have been described (Charles et al., 1997). The three models are: the paternalistic model that assigns decision-making authority solely to the professional, with the patient having no predefined or active role beyond being a recipient of health care; the informed choice model that restricts the healthcare professional’s role to providing information, while the patient independently makes the decision; and the collaborative model of shared decision-making (SDM), where patients and healthcare professionals make decisions together, drawing on both the expertise of the healthcare professional in diagnosing and treatment options and the patient’s preferences regarding the different treatment options (Charles et al., 1997; Elwyn et al., 2000; Stiggelbout et al., 2012). Accordingly, the patient’s role in these models has been described as passive, active, and shared, respectively (Charles et al., 1997; Slade, 2017), corresponding to clinician-led (passive), patient-led (active), and collaborative decision-making (shared) (Slade, 2017).

As patient engagement is increasingly recognized as integral to health care to ensure treatment decisions that benefit the patient and can lead to better outcomes (Marzban et al., 2022), the collaborative model of SDM is often advocated as the ideal model of treatment decision-making in contemporary health care (Aoki et al., 2022; Joseph-Williams et al., 2024). This is particularly relevant in mental health care, where studies have shown that people receiving mental health services wish to be involved in treatment decisions (Hamann et al., 2005) and often desire greater participation than they currently experience (Adams et al., 2007; Dahlqvist et al.,  2015).

Across various medical fields, including mental health services, SDM is supported to some extent by research. Studies have demonstrated positive effects of SDM approaches on outcomes such as patient knowledge and patient involvement, especially when using decision aids to support SDM processes. Decision aids, such as visual cards or option grids, are tools that facilitate SDM practice by presenting treatment options, outlining their pros and cons, and helping patients in reflecting on their treatment preferences in light of the choices they face (Stacey et al., 2024). A recent, updated Cochrane review on decision aids summarizing 209 randomized controlled trials, including 10 trials conducted in mental health services, found that decision aids improve patients’ knowledge, enhance alignment between informed values and care choices, and reduce the proportion of patients who are passive in decision-making. The review also found high-certainty evidence that decision aids increase the accuracy of risk perception and reduce decisional conflict (Stacey et al., 2024).

In mental health services specifically, SDM interventions have demonstrated benefits for both patient involvement and patient activation. A multi-center, cluster randomized trial tested a combined staff and patient training program to promote SDM among inpatients with schizophrenia or schizoaffective diagnoses. The intervention led to a significant increase in patients’ perceived involvement in decision-making (Hamann et al., 2020). A systematic review and meta-analysis of 16 randomized controlled trials on digital SDM interventions in mental health care documented a moderate positive effect on patient activation compared to control conditions (Vitger et al., 2021). A subsequent randomized controlled trial comparing a smartphone-based digital intervention to support SDM in treatment consultation with standard care found a statistically significant effect on patient activation and confidence in communicating with one’s provider in favor of the intervention group (Vitger et al., 2022).

SDM in mental health is also a central part of the recovery paradigm (Matthias et al., 2012; Davidson et al., 2017; Aoki et al., 2022). Recovery was initially defined as a “personal process of changing one’s attitudes, values, feelings, goals, skills, and/or roles” (Anthony, 1993, p. 527), and has since been unfolded as a process that may consist of a variety of elements, including the reestablishment of identity, empowerment, and finding meaning in life (Leamy et al., 2011). Accordingly, recovery-oriented care aims to support people with mental health issues in achieving a meaningful and satisfying life with or without symptoms (Anthony, 1993; Slade et al., 2014; Waldemar et al., 2016). As SDM brings together different forms of expertise in treatment decisions and stresses the personal preferences and values of the patient in treatment planning, SDM processes can play a crucial role in helping people define what matters most to them and thereby supporting their personally-defined recovery goals (Drake et al., 2010; Davidson et al., 2017). For this reason, SDM in mental health care has also been designated as an ethical imperative (Drake & Deegan, 2009), as it upholds the values of choice, self-determination, and empowerment in people with mental health issues (Deegan & Drake, 2006).

Although SDM is supported to some degree by empirical evidence, ethical reasoning, and its alignment with recovery-oriented care, implementing SDM into routine mental health services can be challenging (Slade, 2017). This may be particularly true in inpatient settings, where a dominant focus on treatment adherence to prevent relapses may act as a barrier to SDM by limiting patients’ choices between different treatment options (Verwijmeren & Grootens, 2024). Additionally, the use of coercion in mental health services may preclude the possibility of realizing SDM approaches altogether (Morant et al., 2016; Treichler et al., 2021), unless advance directives are integrated into practice to ensure the treatment preferences of the patient during crisis situations (Farrelly et al., 2016). Moreover, the literature indicates that many mental health professionals consider the SDM model primarily applicable when patients are functioning well and have insight into their mental health condition, thereby often excluding SDM in specific situations or with certain patients (Hamann & Heres, 2014; Haugom et al., 2020).

As stressed by Watson et al. (2022), strategic implementation efforts require an understanding of organizational readiness. In the context of SDM, this includes understanding how mental health professionals themselves approach treatment decisions—whether they prefer passive (i.e., clinician-led), active (i.e., patient-led), or shared decision-making. Since SDM in mental health is part of the recovery paradigm, insight into professionals’ knowledge of and attitudes to the concept of recovery is also essential.

Previous studies have examined mental health professionals’ preferences for clinical decision-making style (e.g., Luciano et al., 2020; Puschner et al., 2016; Rojnic Kuzman et al., 2022), showing, for instance, that being a psychiatrist and working with inpatients increases preferences for a traditional clinician-led decision making, where the patient is passive (Rojnic Kuzman et al., 2022). Other studies have examined professionals’ knowledge of and attitudes to the concept of recovery (Chiba et al., 2017; Cleary & Dowling, 2009; Egeland et al., 2021; Gaffey et al., 2016; Hardy et al., 2022; Nardella et al., 2021; Slade et al., 2015; Walsh et al., 2017). However, to our knowledge, no previous studies have simultaneously examined preferences for decision-making style and professionals’ knowledge of and attitudes to recovery. Understanding this relationship is important in identifying whether professionals who endorse collaborative decision-making are also more aligned with recovery-oriented values. This insight may in turn may help inform future implementation efforts in deciding on different interventions to support SDM and enhance recovery orientation among different professional groups.

Using an exploratory cross-sectional design, we therefore mapped decision-making preferences and knowledge of and attitudes to recovery among mental health professionals working in inpatient and outpatient settings in routine mental health services in one of Denmark’s five regions. To inform SDM implementation, we explored potential differences across professional groups and service settings, and relationships between professionals’ preferred decision-making style and their knowledge of and attitudes to the concept of recovery.

## Methods

### Design

This study draws on survey data examining mental health professionals’ preferences for decision-making style and their knowledge of and attitudes toward recovery. The survey was distributed in November 2022 to all mental health professionals employed within the mental health services in one Denmark’s five regions. Data collection continued through March 2023, and data analysis was conducted from March to June 2023.

The study was approved by the regional data protection authority (Journal number: 22/42525). In accordance with Danish legislation, ethical approval is not required for questionnaire studies; thus, no ethical approval was sought for the present study. Nonetheless, the study was conducted in adherence to the Declaration of Helsinki. A risk assessment was conducted, participant privacy was safeguarded, and data were stored on secure servers. Participants were informed about the study’s purpose, the voluntary nature of their participation, data confidentiality, and their right to withdraw at any time without consequences.

### Setting

In Denmark, people with severe mental illness receive treatment through hospital-based psychiatric services, which include both inpatient and outpatient treatment embedded within each of the five Danish regions. This study focused on the Region of Southern Denmark, where the psychiatric services are organized into a single unified mental health hospital, with eight departments at the time of the study start. Although centrally administered, services are geographically dispersed, with both inpatient and outpatient units located in several areas and towns within the region. Each year, about 40,000 people are in active treatment. Patients receive treatment for varying durations, while 564 inpatient beds are allocated for people with severe mental illness, including those in need of recurrent hospitalizations.

The implementation of SDM has been a strategic priority in the Region of Southern Denmark since 2019. Integrating SDM across all hospitals in the region, including the mental health services, is not seen as optional, but rather as a core element of a broader regional strategy to ensure patient involvement in health care. Within the mental health services specifically, the regional strategy plan for mental health care underscores that “the best possible course of treatment is characterized by people with mental illness being met as equal partners with influence on their own treatment” (The Regional Strategy Plan of Mental Health Care 2020–2025, the Region of Southern Denmark).

Following a generic model for hospital-based implementation of SDM (Steffensen et al., 2023), each rollout is carried out through a systematic process of education and training. A central part of the SDM model in the region is to train designated clinicians in each clinical department to become SDM teachers of the department. This training is a two-day course led by well-educated SDM trainers, after which the teachers are responsible for educating and training fellow clinicians in their department to apply SDM in treatment consultations. In the model, the leaders of the departments are also trained in SDM and in leadership during the SDM implementation. Each phase of the implementation is characterized by structured planning, execution, and follow-up, also involving developing, testing, and implementing decision aids (Steffensen et al., 2023).

## Participants and Procedures

At the time of study start, 3,128 individuals were employed in the region’s mental health services. This workforce included 996 nurses and 320 doctors. Other key professionals included psychologists (*N* = 245), occupational and physical therapists (*N* = 158), social workers (*N* = 100), social and health care assistants (*N* = 642) and peer support workers (*N* = 52). Approximately 60% of the staff worked in inpatient settings, while the remaining 40% worked in outpatient settings. Women accounted for 80% of the workforce.

The survey was conducted online via REDCap hosted on the secure server of Odense Patient Data Explorative Network (OPEN) in the Region of Southern Denmark (Harris et al., 2009, Harris et al., 2019). An email containing an open survey link was sent from REDCap to the central mailboxes of the eight mental health departments. Each department was responsible for distributing the link to their staff. The email also included a cover letter stating the purpose of the study and clarifying that only staff with direct patient contact were eligible for participation.

Participation was voluntary and anonymous. Participants provided informed consent by reading study information and clicking on the link to the online survey.

## Measures

### Demographic Characteristics

Participants reported their profession, work setting (inpatient or outpatient), age, gender, and length of employment in mental health care. The survey also included two items related to SDM: whether the participant had received formal SDM training (yes/no) and whether a decision aid to support SDM practices was available in their team or unit (yes/no). These items were included to explore potential associations between SDM training and/or having a decision aid and both decision-making style and professionals’ knowledge about and attitudes to recovery.

## Clinical Decision-Making Style

To measure clinical decision-making style, we used the standardized staff version of the Clinical Decision Making Style (CDMS-S), developed as part of a large European study on clinical decision-making across several European countries (Puschner et al., 2010, Puschner et al., 2013). It consists of two sub-scales with 21 items in total.

The Participation in Decision Making (PD) subscale consists of 15 items. The first six items assess general preferences for decision-making in routine mental health services. These are rated on a Likert scale from 0 (strongly disagree) to 4 (strongly agree), with four items reverse-coded. The remaining nine items address preferences for decision-making in relation to three clinical vignettes (work, medication side effects, and medication in general). These items are rated on a scale from 0 (me/the therapist alone) to 4 (the patient alone). The PD score is calculated as the prorated mean of all 15 items, provided that at least 12 items have been completed. Scores range from 0 to 4, with higher scores indicating a stronger preference by the clinician for active service-user participation in decision-making. Based on the average score, responses are categorized into one of three decision-making styles: passive (< 1.5), shared (1.5–2.5) or active (> 2.5), reflecting the respondent’s preferred level of patient involvement in clinical decision-making (Puschner et al., 2013).

The Information (IN) subscale consists of six items, of which five items are included in the score (see Puschner et al., 2013). Responses are rated on a Likert scale from 0 (strongly disagree) to 4 (strongly agree). The IN score is calculated as the prorated mean of the items and can be calculated when at least four of the five items have been rated. Higher scores indicate a greater preference of the professional for providing information to the patient.

Evaluation of the CDMS-S has shown good psychometric properties (Puschner et al., 2013: Rojnic Kuzman et al., 2022). The internal reliability for the CDMS-S PD in this study was good, with a Cronbach’s alpha reliability coefficient of α = 0.82. The internal reliability on the CDMS-S IN was lower, with α = 0.69.

### The Recovery Knowledge Inventory (RKI)

The Recovery Knowledge Inventory (RKI) was used to assess mental health professionals’ knowledge of and attitudes to recovery. As recovery may not be readily defined in objective terms, the RKI captures a blend of conceptual understanding and attitudinal orientation toward the concept of recovery, as defined in contemporary mental health policy and practice (Bedregal et al., 2006).

RKI was originally developed as a 36-item self-report instrument. After being tested, it was reduced to a measure consisting of 17 items, but after consulting the literature and stakeholders, three of the discarded items were restored, resulting in a scale with 20 items (Bedregal et al., 2006).

The 20 items of RKI are rated on a five-point Likert scale ranging from 1 (strongly disagree) to 5 (strongly agree), with several items reverse-coded. A total score is calculated as the mean across all items, with higher scores reflecting greater knowledge and positivity towards recovery-oriented approaches.

The RKI was developed to assess four conceptual domains: (a) roles and responsibilities in recovery, (b) non-linearity of the recovery process, (c) roles of self-definition and peers in recovery, and (d) expectations regarding recovery. However, studies have called into question this four-factor structure (Chiba et al., 2017; Happell et al., 2015; Lakshman et al., 2023). Due to these concerns, only the total RKI score was used. The internal reliability in this study was good, with a Cronbach’s α = 0.81.

## Analyses

Data analyses were conducted in Stata version 18. Descriptive statistics were reported as counts and percentages for categorical variables, and means and standard deviations for continuous variables. Because the study was explorative and hypothesis-generating, our analytical strategy was designed to map patterns of variation across different contexts. We used a series of univariate analyses to present the immediate patterns observed in the data. To allow for multiple testing, and to reduce the risk of type I error, all test were two sided with a significance threshold of *p* < 0.001.

To explore associations between participants’ characteristics, their preferences for decision-making styles (CDMS-S PD) and information-sharing (CDMS-S IN), and their knowledge of and attitudes to recovery (RKI), we conducted a series of one-way ANOVAs. For each analysis, we reported test statistics with 95% confidence intervals, and effect sizes (Cohen’s *d* for binary variables and eta squared [η²] for multi-category variables).

To examine the relationship between decision-making style (categorized as passive, shared, or active) and participant characteristics, chi-square tests were used. This categorical breakdown complements the continuous analyses and aims to provide a more interpretable picture of group differences in preferences for decision-making style.

Finally, to test whether knowledge of and attitudes to recovery (RKI) differed between participants categorized as preferring a passive, shared, or active decision-making style, we conducted a one-way ANOVA.

## Results

### Study Sample and Participant Characteristics

**Fig. 1:**
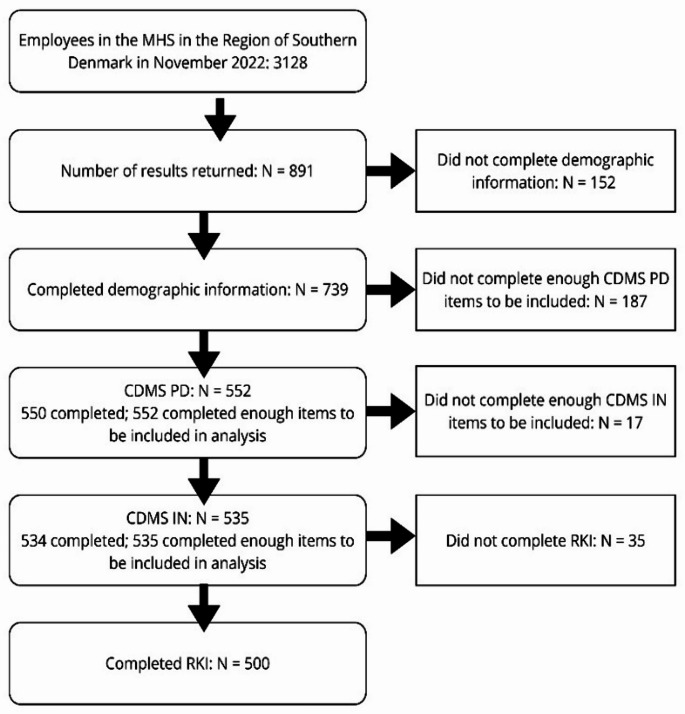
Flowchart of participant inclusion

Among 3,128 employees, 891 (28.5%) questionnaires were returned. Of these, 152 were excluded due to missing background information. Of the remaining participants, 552 (17.6%) completed enough items to be included in the analyses on preferences for decision-making style, 535 (17.1%) completed enough items to be included in analysis of CDMS-S IN subscale, and 500 (16.0%) participants completed RKI; see flowchart in Fig. 1.

The characteristics of the 552 participants who completed the CDMS-S PD subscale are shown in Table 1, along with mean scores on the CDMS-S (PD and IN) and RKI. Nearly half of the participants were between 31 and 50 years or older and the majority of the participants were women, reflecting the overall demographic composition in the actual regional workforce. Participants represented a range of professional backgrounds and work settings. Nurses and peer support workers were slightly overrepresented relative to their proportion in the regional workforce, and responses from outpatient staff were also slightly overrepresented. One in five participants had received training in SDM and 15.6% reported that they had a decision-support tool available in their team or unit.

**Table 1:** Sociodemographic characteristics and mean scores on participation, information sharing, and measures of knowledge of and attitudes to recovery

Sample Characteristics	Participants (*N* = 552)		Clinical Decision Making Style – Staff Questionnaire				Recovery Knowledge Inventory	
			CDMS-S PD (*N* = 552)		CDMS-S IN (*N* = 535)		RKI total (*N* = 500)	
	*N*	%	M	SD	M	SD	M	SD
Sex								
Female	447	81	2.07	0.46	2.95	0.54	3.43	0.39
Male			2.05	0.49	3.02	0.51	3.24	0.36
Age								
≤ 30 years	47	8.5	1.99	0.45	3.07	0.48	3.35	0.39
31–50	272	49.3	2.06	0.46	2.94	0.48	3.41	0.38
≥ 51	233	42.2	2.08	0.48	2.96	0.61	3.38	0.41
Profession								
Doctor	52	9.4	2.09	0.35	2.78	0.56	3.37	0.39
Nurse	217	39.3	2.15	0.44	2.89	0.57	3.46	0.40
Social health and care assistant	100	18.1	1.82	0.53	3.06	0.49	3.24	0.31
Peer worker	22	4.0	2.13	0.53	3.03	0.56	3.58	0.57
Therapist (physiotherapist or occupational therapist, psychologist)	86	15.6	2.12	0.38	2.95	0.42	3.40	0.33
Social worker, pedagogues or other	75	13.6	2.01	0.48	3.17	0.54	3.37	0.44
Mental health work experience								
< 5 years	182	33.0	1.90	0.48	3.03	0.48	3.30	0.38
5 to 12 years	131	23.7	2.03	0.48	3.02	0.56	3.42	0.39
> 12 years	239	43.3	2.21	0.39	2.89	0.55	3.45	0.40
Setting								
Inpatient	274	49.6	1.92	0.50	3.00	0.53	3.32	0.41
Outpatient	246	44.6	2.21	0.36	2.91	0.51	3.46	0.36
Other	32	5.8	2.16	0.47	3.08	0.78	3.52	0.42
Had received training in shared decision making								
Yes	112	20.3	2.14	0.46	2.91	0.54	3.45	0.43
No	440	79.7	2.04	0.46	2.98	0.54	3.38	0.38
Had a Decision Helper available in ward/team								
Yes	86	15.6	1.99	0.47	2.90	0.48	3.29	0.37
No	466	84.4	2.08	0.46	2.97	0.55	3.42	0.40

Note. CDMS-S = Clinical Decision Making Style – Staff Questionnaire; PD = Participation in Decision Making Subscale; IN = Information Subscale; RKI = Recovery Knowledge Inventory; M = mean; SD = standard deviation. Values for sociodemographic characteristics reflect absolute counts (N) and percentages (%). Means and standard deviations are presented for each outcome measure by subgroup. Sample sizes vary due to item-level missingness: CDMS-S PD (*N* = 552), CDMS-S IN (*N* = 535), and RKI total score (*N* = 500)

### Associations between Participant Characteristics, Decision-Making Styles, and Knowledge of and Attitudes To Recovery

Full results for the associations between participant characteristics, preferences for decision-making style and information-sharing (CDMS-S PD and IN), and knowledge of and attitudes to recovery (RKI), including test statistics with confidence intervals and effect sizes, are presented in Table 2. The most consistent exploratory patterns (*p* < 0.001) were observed across professional groups, with differences for all three outcomes (means and standard deviations by group can be seen in Table 1). Amongst professions, nurses had the highest mean levels on participation preference, while social and health care assistants scored lowest on both participation preference and knowledge of and attitudes to recovery. Peer workers had the highest recovery knowledge scores.

Further exploratory differences were observed for work experience, where participants with longer experience in mental health care reported higher levels of participation preference. Patterns also emerged for recovery knowledge and attitudes by sex, with women reporting higher scores than men, and by service setting, where outpatient and ‘other’ staff reported both higher participation scores and recovery knowledge compared with inpatient staff.

**Table 2:** Associations between participant characteristics and scores on participation, information sharing, and knowledge of and attitudes to recovery

Variables	CDMS-S						RKI		
	Participation Score (CDMS-S PD) (*N* = 552)			Information Score (CDMS-S IN) (*N* = 535)			RKI total (*N* = 500)		
	Test-statistic	95%-CI	Effect size	Test-statistic	95%-CI	Effect size	Test-statistic	95%-CI	Effect size
Sex	*F*(1, 550) = 0.17	[0.03, 172.08]	*d =* 0.04	*F*(1, 533) = 1.26	[0.25, 1277.20]	*d* = 0.12	*F*(1, 498) = 18.44*	[3.65, 18760.33]	*d* = 0.49
Age group	*F*(2, 549) = 0.62	[0.17, 24.53]	*η*^*2*^ = 0.00	*F*(2, 532) = 1.07	[0.29, 42.13]	*η*^*2*^ = 0.00	*F*(2, 497) = 0.60	[0.16, 23.89]	*η*^*2*^ = 0.00
Profession	*F*(5, 546) = 8.02*	[3.10, 48.35]	*η*^*2*^ = 0.07	*F*(5, 529) = 4.89*	[1.89, 29.45]	*η*^*2*^ = 0.04	*F*(5, 494) = 5.16*	[1.99, 31.13]	*η*^*2*^ = 0.05
Work experience	*F*(2, 549) = 25.5*	[6.87, 1007.12]	*η*^*2*^ = 0.08	*F*(2, 532) = 4.10	[1.10, 161.92]	*η*^*2*^ = 0.02	*F*(2, 497) = 6.81	[1.83, 268.75]	*η*^*2*^ = 0.03
Setting	*F*(2, 549) = 27.2*	[7.33, 1075.46]	*η*^*2*^ = 0.09	*F*(2, 532) = 2.25	[0.60, 88.74]	*η*^*2*^ = 0.01	*F*(2, 497) = 8.95*	[2.41, 353.62]	*η*^*2*^ = 0.03
Prior training SDM	*F*(1,550) = 3.96	[0.78, 4025.30]	*d* = 0.21	*F*(1, 533) = 1.36	[0.27, 1385.35]	*d* = 0.13	*F*(1. 498) = 2.28	[0.45, 2317.98]	*d* = 0.17
SDM tool in team/ward	*F*(1, 550) = 2.58	[0.51, 2621.36]	*d* = 0.19	*F*(1, 533) = 1.21	[0.24, 1227.04]	*d* = 0.13	*F*(1, 498) = 7.11	[1.41, 7233.13]	*d* = 0.33

Note. CDMS-S = Clinical Decision Making Style – Staff Questionnaire; PD = Participation in Decision-Making; IN = Information-Sharing; RKI = Recovery Knowledge Inventory; SD = standard deviation; CI = confidence interval; d = Cohen’s d; η² = eta squared. Observed power was computed using α = 0.001.* = *p* < 0.001. Effect sizes were calculated as Cohen’s d for binary variables and η² for multi-category variables

### Distribution of Decision-Making Styles according to Participant Characteristics

To supplement the analyses of mean differences, CDMS-S PD scores were categorized into decision-making styles (passive, shared, or active). Chi-square tests were used to examine whether the distribution of decision-making styles varied across participant characteristics. As shown in Table 3, significant differences were observed for service setting, work experience, and profession, while no significant variation was found by gender, age, or prior SDM training.

Across the sample, SDM was the most frequently preferred decision-making style. Participants with more experience and those working in outpatient settings were more often categorized as preferring either shared or active decision-making styles, whereas those with less experience or working in inpatient settings were more often categorized as preferring a traditional clinician-led decision-making style, in which the patient is passive.

**Table 3:** Decision-making style distribution by participant characteristics (*N* = 534)

	Decision-Making Style Classified According to the CDMS-S PD							
	Passive		Shared		Active		Test-statistic	p-value
	N	%	N	%	N	%		
All	66	12.0	400	72.4	68	15.5		
Sex							χ2 (2) = 1.06	0.588
Female	51	11.4	328	73.4	68	15.2		
Male	15	14.3	72	68.6	18	17.1		
Age							χ2 (4) = 4.79	0.309
< 30	7	14.9	37	78.7	3	6.4		
31-50	32	11.8	200	73.5	40	14.7		
≥ 51	27	11.6	163	70.0	43	18.5		
Profession							χ2 (10) = 45.36	< 0.001
Doctor	0	0.0	46	88.5	6	11.5		
Nurse	17	7.8	154	71.0	46	21.2		
Social health and care assistant	28	28.0	62	62.0	10	10.0		
Peer worker	3	13.6	15	68.2	4	18.2		
Therapist (physiotherapist or occupational therapist, psychologist)	7	8.1	70	81.4	9	10.5		
Social worker, pedagogues or other	11	14.7	53	70.7	11	14.7		
Mental health work experience							χ2 (4) = 39.92	< 0.001
< 5 years	39	21.4	128	70.3	15	18.2		
6 to 12 years	18	13.7	94	71.8	19	14.5		
>12 years	9	3.8	178	74.5	52	21.8		
Setting							χ2 (4) = 51.34	< 0.001
Inpatient	58	21.2	188	68.6	28	10.2		
Outpatient	5	2.0	190	77.2	51	20.7		
Other	3	9.4	22	68.8	7	21.9		
Had received training in shared decision making							χ2 (2) =2.72	0.256
Yes	9	8.0	82	73.2	21	18.8		
No	57	13.0	318	72.3	65	14.8		
Had a Decision Helper available in ward/team							χ2 (2) = 4.29	.117
Yes	11	12.8	68	79.1	7	8.1		
No	55	11.8	332	71.2	79	17.0		

Note. CDMS-S = Clinical Decision Making Style – Staff Questionnaire. Decision making style defined according to CMDS-S PD score: Passive (CMDS-S PD score <1.5), Shared (CMDS-S PD score 1.5-2.5), active (CMDS-S PD score >2.5). χ² tests were used to examine the association between participant characteristics and decision-making style classification. Percentages are column percentages

Professional role was also associated with decision-making preferences. For example, no doctors were classified as preferring a passive decision-making style, whereas amongst those with less experience or working in inpatient settings, a higher proportion were classified as preferring a passive style.

### Differences in Knowledge of and Attitudes to Recovery by Preferred Decision-Making Style

Next, we examined whether professionals’ preferences for decision-making styles were associated with differences in knowledge of and attitudes to recovery. As shown in Table 4, mean RKI scores followed a progressive pattern, increasing from passive to shared, and then to active decision-making styles. These findings suggest that professionals who prefer active or shared decision-making styles report higher levels of knowledge of and positive attitudes to recovery than those who prefer a passive style.

**Table 4:** Comparisons of knowledge of and attitudes to recovery score between Decision-Making styles (*N* = 500)

RKI across Decision-Making Styles						Mean difference (95%-CI) of RKI total scale between					
Passive(*N* = 54)		Shared (*N* = 366)		Active (*N* = 80)		Shared vs. passive		Active vs. passive		Active vs. shared	
M	SD	M	SD	M	SD	Mdiff	CI	Mdiff	CI	Mdiff	CI
3.05	0.31	3.39	0.36	3.68	0.40	0.34	[0.21, 0.47]	0.63	[0.48, 0.79]	0.29	[0.18, 0.40]

Note. RKI = Recovery Knowledge Inventory. Mdiff = mean difference. CI = confidence intervals. Observed power was computed using α = 0.001. All mean differences were statistically significant at *p* < 0.001

## Discussion

This study investigated mental health professionals’ preferred decision-making styles and their knowledge of and attitudes to recovery. Consistent with previous European studies (Luciano et al., 2020; Rojnic Kuzman et al., 2022), the majority of participants reported a preference for SDM. However, our findings also indicate that this preference may not be uniformly distributed across service settings, levels of clinical experience, or professional roles.

The findings may reflect distinct clinical responsibilities, educational backgrounds, or professional cultures (Pappa et al., 2021). However, given the exploratory nature of these comparisons and the lack of systematic pairwise testing, the findings should be considered hypothesis-generating rather than conclusive. Nevertheless, such potential differences warrant further investigation, as they may suggest a need for differentiated implementation strategies tailored to specific professional roles and practice settings. Future studies could further investigate the observed variations across professional groups, clinical experience, and setting, considering potential confounders.

Compared to previous international studies, the overall knowledge of and attitudes to recovery score in our sample was somewhat higher (Chiba et al., 2017; Egeland et al., 2021; Hardy et al., 2022; Slade et al., 2015). Contributing to the literature, we found a positive association between knowledge of and attitudes to recovery and professionals’ preferences for decision-making style. Participants who preferred a shared or active style scored higher on the RKI compared to those who preferred a passive, clinician-led style. These differences were statistically significant and may suggest a meaningful relationship between endorsement of shared- or active decision-making and recovery-oriented values. While the cross-sectional nature of the study precludes causal inference, this association may indicate that professionals who are more familiar with or committed to recovery principles may also be more inclined to engage patients in decision-making processes. This interpretation resonates with previous research suggesting that shared decision-making is closely tied to a broader epistemological stance on the distribution of expertise, power, and responsibility in care (Leamy et al., 2011; Slade, 2017).

The findings could have practical implications for workforce development and implementation strategies. If high knowledge of and positive attitudes to recovery and preference for SDM tend to co-occur, training programs targeting one may also strengthen the other. However, the direction and nature of this relationship remains unclear. Future longitudinal or interventional studies are needed to determine whether training in recovery-oriented practice enhances shared decision-making preferences—or whether promoting SDM may deepen professionals’ engagement with recovery values.

Despite the strategic emphasis on SDM in the region, only one in five participants in our sample reported having received formal training in SDM at the time of the study conduction, and access to structured decision aids was limited. Although this may partly reflect that the study was conducted at an early stage of the implementation, it nevertheless point to a potential gap between policy ambitions and practice. A further strengthening of the organizational support structures for the implementation of SDM, such as training programs and decision aids, may be important in closing this gap.

As part of future implementation efforts to transform preferences for SDM and recovery knowledge and attitudes into practice, peer support workers may offer a valuable and underutilized resource. Although no formal comparisons were made across professions, peer support workers reported strong knowledge of and attitudes to recovery, and a clear preference for SDM. Their unique position and lived experience could enable them to act as cultural brokers or co-facilitators in training programs to foster recovery-oriented, collaborative practices (Jensen et al., 2025).

Finally, we also note that our findings point to a graded pattern that invites further reflection: while both shared and active decision-making styles were associated with higher knowledge of and attitudes to recovery, those preferring an active, informed-choice style scored significantly higher than those preferring a shared style. One possible interpretation is that active decision-making preferences may be particularly salient among professionals who see recovery as a process that emphasizes personal agency, autonomy, and self-determination. Importantly, such preferences do not stand in opposition to SDM but rather expand its scope. Meaningful involvement and SDM are also about matching the needs and preferences of the patient—including the preferences of the individual patient to lead, share, or even defer decision-making. Future research could use longitudinal and observational studies to explore how preferences relate to practice and how recovery values are enacted in real-world settings.

### Limitations

The study has several limitations. As already noted, the cross-sectional and explorative design limits causal inference and generalizability. We also note that the findings reflect reported preferences and attitudes, not actual clinical behavior, and that self-report data may be influenced by social desirability, especially in relation to SDM. Prior studies comparing clinician and patient reports of SDM have shown notable discrepancies, with professionals often rating the degree of SDM more favorably than do patients (Drivenes et al., 2020; Pappa et al., 2021). In addition, recent qualitative findings suggest that although mental health professionals advocate SDM as their favored approach, in reality they may adopt a more directive or informative approach, offering advice or instructions, rather than offering treatment choice in an SDM practice (Gurtner et al., 2024). In this regard, participants in our study may have reported socially desirable preferences rather than their actual beliefs. The relatively low response rate and the regional sample further limit representativeness. While the RKI allowed for comparison with other studies, concerns have been raised about its psychometric robustness.

Finally, as the aim of the present study was to map observed patterns of variations across participants, potential confounding issues were not considered. While findings should be interpreted with caution due to the exploratory nature of our study, the use of unadjusted significance thresholds, and the lack of post hoc comparisons between specific groups, we recommend that future studies apply multivariable approaches to build on our exploratory insights and examine whether the observed patterns hold after adjustment for potential confounders.

## Conclusion

This study highlights variations in decision-making preferences among mental health professionals and their association with knowledge of and attitudes to recovery. While SDM was widely favored, preferences differed across levels of clinical experience, professions, and settings. The link between SDM preferences, and knowledge of and attitudes to recovery, points to a potential leverage for implementation efforts—but further research is needed to understand how these preferences translate into everyday clinical practice.

## Data Availability

The datasets used and/or analyzed during the current study are available from the corresponding author on reasonable request, provided that the departments approve of disclosure.
